# High SAA1 Expression Predicts Advanced Tumors in Renal Cancer

**DOI:** 10.3389/fonc.2021.649761

**Published:** 2021-05-18

**Authors:** Sen Li, Yongbiao Cheng, Gong Cheng, Tianbo Xu, Yuzhong Ye, Qi Miu, Qi Cao, Xiong Yang, Hailong Ruan, Xiaoping Zhang

**Affiliations:** ^1^ Department of Urology, Union Hospital, Tongji Medical College, Huazhong University of Science and Technology, Wuhan, China; ^2^ Institute of Urology, Union Hospital, Tongji Medical College, Huazhong University of Science and Technology, Wuhan, China; ^3^ Key Lab for Biological Targeted Therapy of Education Ministry and Hubei Province, Union Hospital, Tongji Medical College, Huazhong University of Science and Technology, Wuhan, China

**Keywords:** SAA1, diagnosis, prognosis, biomarker, renal cancer

## Abstract

Renal cell carcinoma (RCC) is the most frequent malignant tumor of the kidney. 30% of patients with RCC are diagnosed at an advanced stage. Clear cell renal cell carcinoma (ccRCC) is the most common pathological subtype of RCC. Currently, advanced ccRCC lacks reliable diagnostic and prognostic markers. We explored the potential of SAA1 as a diagnostic and prognostic marker for advanced ccRCC. In this study, we mined and analyzed the public cancer databases (TCGA, UALCAN and GEPIA) to conclude that SAA1 was up-regulated at mRNA and protein levels in advanced ccRCC. We further found that hypomethylation of SAA1 promoter region was responsible for its high expression in ccRCC. Receiver operating characteristic curve (ROC) indicated that high SAA1 levels could distinguish advanced ccRCC patients from normal subjects (p < 0.0001). Kaplan-Meier curve analysis showed that high SAA1 levels predicted poor overall survival time (p < 0.0001) and poor disease-free survival time (p = 0.0003). Finally, the functional roles of SAA1 were examined using a si-SAA1 knockdown method in RCC cell lines. Our results suggest that SAA1 may possess the potential to serve as a diagnostic and prognostic biomarker for advanced ccRCC patients. Moreover, targeting SAA1 may represent as a novel therapeutic target for advanced ccRCC patients.

## Introduction

In the United States, renal cancer represents respectively the 6th and 8th most common malignancy in men and in women, accounting for about 3% of cancer deaths (1). Cancer statistics show that approximately 73,820 new cases of renal cancer and expected 14,770 deaths happened in the United States in 2019 (1). Clear cell renal cell carcinoma (ccRCC) is clinically divided into localized ccRCC (L-ccRCC), locally advanced ccRCC (LA-ccRCC), and metastatic ccRCC (M-ccRCC). L-ccRCC and LA-ccRCC can achieve clinical curative effect through nephron-sparing surgery or nephrectomy. M-ccRCC requires comprehensive medical treatment, including cytoreductive nephrectomy, molecular targeted therapy and immunotherapy (2–6). Although surgical treatment, targeted therapy and immunotherapy have acquired great progress in recent years, there are still many patients with advanced ccRCC or M-ccRCC die from this disease due to treatment tolerance (7, 8). Therefore, the progress and metastatic mechanisms of locally advanced ccRCC and M-ccRCC are the primary tasks of current research.

SAA1 protein belongs to a member of the serum amyloid A family of apolipoproteins. SAA1 is a major acute-phase protein whose expression is upregulated when the body is stressed by inflammation and tissue damage (9). In addition, SAA1 expression also can be induced following surgery or in advanced malignancies (10). SAA1 also plays a critical role in high-density lipoprotein metabolism and cholesterol homeostasis (11, 12). Extensive literatures have reported that SAA1 could contribute to cancer development and accelerate tumor progression and distant metastasis (10). For example, SAA1 enhances plasminogen activation to promote colon cancer progression (13). SAA1 may interact with the extracellular matrix to change its affinity to cells, leading to cell metastasis (14). In addition, a large number of studies have confirmed the positive correlation between SAA1 concentrations and tumor stage (15, 16). In a sample of 233 different tumor patients, higher SAA1 levels appeared in more advanced tumor patients (17). Moreover, the up-regulation of SAA1 could be used as a biomarker for a variety of malignant tumors (18–20). Most importantly, serum SAA1 was identified as a biomarker of distant metastases but not as an early tumor marker in RCC patients (21). However, the diagnostic and prognostic potential of SAA1 at the tumor tissue level and its biological function in ccRCC have not been reported.

In this study, we were committed to exploring the diagnostic and prognostic value of SAA1 in ccRCC, especially in advanced ccRCC, and strived to explore the therapeutic potential of targeting SAA1.

## Materials and Methods

### Data Download

We downloaded the GSE11151 (22), GSE6344 (23) and GSE781 (24) datasets from the GEO database (https://www.ncbi.nlm.nih.gov/geo/), which is a public and shared cancer database. We took the top ten up-regulated genes in these three datasets to analyze the intersection genes.

### Data Processing

The differentially expressed genes (DEGs) of GSE11151, GSE6344 and GSE781 datasets were identified using GEO2R (https://www.ncbi.nlm.nih.gov/geo/geo2r/), an available online analysis software for the GEO database, which was dependent on R language programming. According to the criteria of logFC ≥2 or logFC ≤-2 and adjusted p value < 0.05, DEGs from the three datasets were identified. We used the Wayne diagram to screen the intersection of three datasets with the top 10 up-regulated DEGs.

### TCGA Database

The mRNA expression data of SAA1 in ccRCC tissues and para-cancer tissues and clinicopathological features including gender, age, T stage, tumor grade, M stage, N stage, histopathological stage, overall survival (OS) were downloaded from TCGA-KIRC datasets (https://xenabrowser.net/heatmap/). Kaplan-Meier curves and ROC curves were analyzed using mRNA levels from TCGA-KIRC dataset.

### UALCAN Online Analysis

SAA1 mRNA, protein expression and promoter methylation levels were evaluated using the UALCAN online analysis software (http://ualcan.path.uab.edu/index.html) (25).

### GEPIA

SAA1 mRNA, overall survival and disease-free survival were also evaluated using GEPIA online analysis software (http://gepia.cancer-pku.cn/) (26).

### ROC Curves Analysis of SAA1

The potential diagnostic value of SAA1 was evaluated using the receiver operating characteristic (ROC) curves by Graphpad Prism software.

### Cell Culture and Transfection

Human RCC cell lines 786-O, ACHN, A-498, Caki-1 and normal renal tubular epithelial cells HK-2 were obtained from ATCC. OS-RC-2 cell line was a gift from the Department of Urology of Wuhan Tongji Hospital. All cell lines were cultured in DMEM medium containing 1% penicillin-streptomycin and 10% FBS. Small interfering RNA (si-RNA) against SAA1 and corresponding negative control (si-NC) were purchased from Ribobio Biological Co., Ltd. (Guangzhou, China). si-SAA1 sequences and si-NC sequences were transfected into cells using Lipofectamine 2000 reagent.

### Immunohistochemistry (IHC), Transwell Migration and Invasion, and Western Blotting (WB) Assays

ccRCC tissues and adjacent normal tissues were fixed in 10% formalin, dehydrated, and embedded in paraffin sequentially. The paraffin sections were incubated with anti-SAA1 antibody overnight at 4°C. Transwell migration and invasion were performed using 24-well transwell chambers. The specific details of these experiments were previously described (27).

### ccRCC Tissue Samples

We collected ccRCC tissues and adjacent normal tissues of 30 case patients who were subjected to partial nephrectomies or nephrectomies at Wuhan Union Hospital between 2018 and 2019. All patients had signed an informed consent form. This study was approved by the Ethics Committee of Huazhong University of Science and Technology.

### Statistical Analysis

SPSS statistical software and Graphpad Prism 7.0 were used for statistical analysis. The SAA1 mRNA levels were analyzed among different clinicopathological features of ccRCC using the Mann-Whitney test. Pearson’s chi-square test was used to analyze the correlation between SAA1 expression levels and clinicopathological features of ccRCC. The ROC curve was used to distinguish ccRCC patients and obtain the area under the curve (AUC). The Kaplan-Meier curve was used to analyze the relationship between the expression level of SAA1 and the overall survival and progression-free survival of ccRCC patients. Each group of data is presented as mean ± SD. The p value<0.05 was considered statistically significant.

## Results

### Screening and Prognostic Analysis of Up-Regulated Target Genes in ccRCC Patients

**Figure 1:**
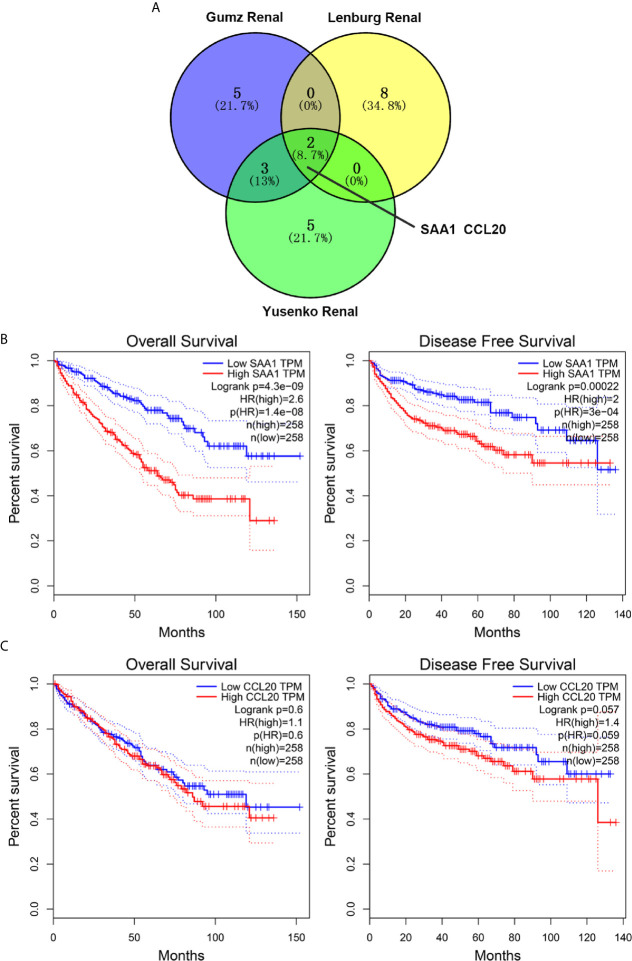
Screening and prognostic analysis of up-regulated target genes in ccRCC patients. **(A)** Wayne diagram showing top ten up-regulated genes in three public databases. **(B)** Kaplan Meier curve analysis for the effect of SAA1 expression on the prognosis of ccRCC patients. **(C)** Kaplan Meier curve analysis for the effect of CCL20 expression on the prognosis of ccRCC patients.

By analyzing three public cancer datasets, we found two intersection genes, SAA1 and CCL20, in these three datasets (**Figure 1A**). Next, we analyzed the correlation between the expression levels of these two genes and the overall and disease-free survival of patients with ccRCC. We found that only SAA1 expression was associated with prognosis in patients with ccRCC and high SAA1 expression indicated a worse prognosis (**Figures 1B, C**).

### SAA1 Is Highly Expressed and Predicts High Tumor Stage in Advanced and Metastatic ccRCC Patients

**Figure 2:**
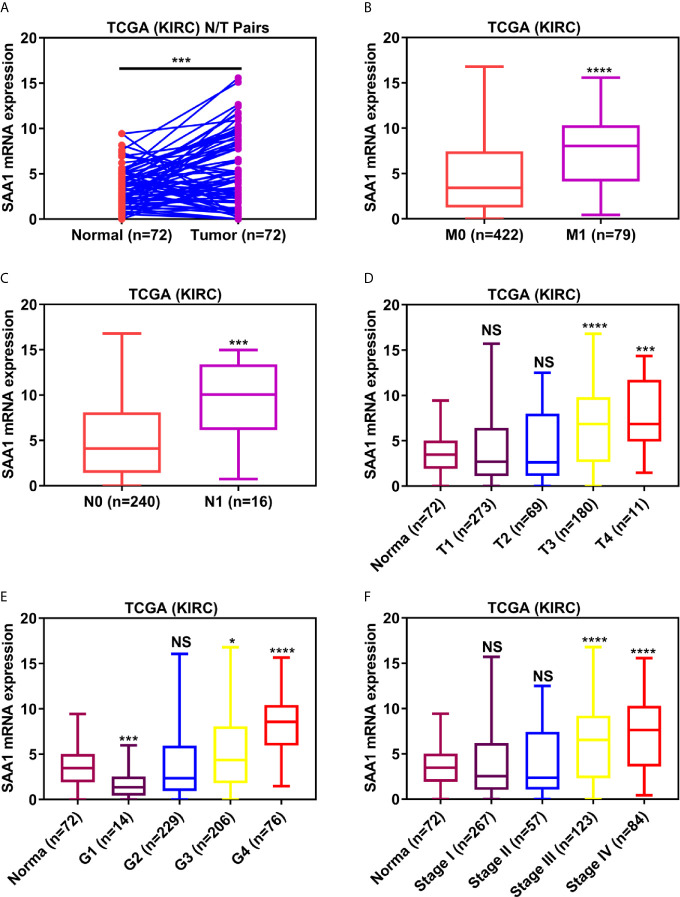
Analysis of SAA1 mRNA expression using TCGA database. **(A)** The mRNA expression levels of SAA1 were up-regulated in ccRCC samples, which were downloaded from TCGA-KIRC database containing 72 paired ccRCC samples. The expression levels of SAA1 mRNA were compared in various tumor stages: **(B)** M stage, **(C)** N stage, **(D)** T stage, **(E)** tumor grade, **(F)** TNM stage. (****P <0.0001, ***P <0.001, *P <0.05, NS means no significance, compared with the respective control).

**Figure 3:**
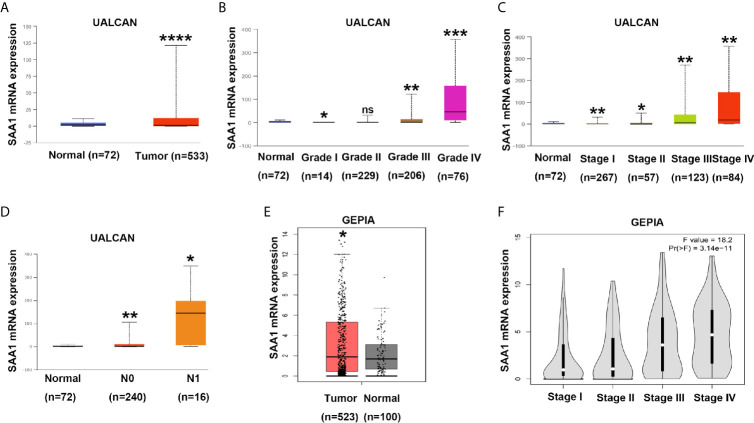
Analysis of SAA1 mRNA expression using UALCAN and GEPIA database. **(A)** The mRNA expression levels of SAA1 were up-regulated in ccRCC samples from UALCAN database. **(B)** The expression levels of SAA1 mRNA were compared in tumor grade from UALCAN database. **(C)** The expression levels of SAA1 mRNA were compared in TNM stage from UALCAN database. **(D)** The expression levels of SAA1 mRNA were compared in N stage from UALCAN database. **(E)** The mRNA expression levels of SAA1 were up-regulated in ccRCC samples from GEPIA database. **(F)** The expression levels of SAA1 mRNA were compared in TNM stage from GEPIA database. (****P<0.0001, ***P<0.001, **P<0.01, *P<0.05, NS means no significance, compared with the respective control).

**Figure 4:**
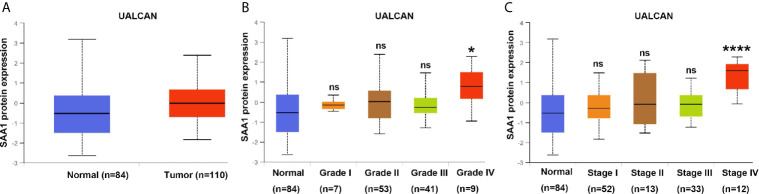
Analysis of SAA1 protein expression using UALCAN database. **(A)** The protein expression analysis of SAA1 in ccRCC samples from UALCAN database. **(B)** The protein expression levels of SAA1 were compared in tumor grade from UALCAN database. **(C)** The protein expression levels of SAA1 were compared in TNM stage from UALCAN database. (****P<0.0001, *P<0.05, NS means no significance, compared with the respective control).

To verify the reliability of the above three studies, we analyzed the expression of SAA1 and its association with clinical pathological parameters in the TCGA database. As shown in **Figure 2A**, SAA1 expression in tumor tissues is upregulated at mRNA levels. Next, we analyzed the mRNA expression levels of SAA1 against T stage, N stage, M stage, Grade classification, histopathological stage and its correlation with these clinicopathological parameters in patients with ccRCC (**Figures 2B–F** and **Table 1**). Our analysis found that the mRNA expression of SAA1 was positively correlated with these clinicopathological parameters and that the mRNA expression of SAA1 increased significantly in higher tumor stages but did not increase or show a downward trend in early tumor stages (**Figures 2B–F**). These results suggested that elevated mRNA expression of SAA1 was predictive of advanced tumor stages. To make our study more precise, we also analyzed the expression of SAA1 in the UALCAN and GEPIA online tumor database websites. As shown in **Figures 3A–F**, high SAA1 expression mainly occurred in patients with advanced and metastatic ccRCC, which were consistent with the analysis results of the TCGA database. Similarly, we also analyzed the expression of SAA1 at the protein level, and the results showed that SAA1 protein expression increased significantly in patients with advanced ccRCC (**Figures 4A–C**). The above results indicate that SAA1 is highly expressed at mRNA and protein levels and predicts high stage risk in patients with advanced and metastatic ccRCC.

**Table 1:** Association of SAA1 mRNA expression with clinicopathological parameters in ccRCC patients.

Parameters		Number	SAA1 mRNA expression		
			Low (n=124)	High (n=124)	P value
Age	< 60	104	51	53	
	>= 60	144	73	71	0.797
Gender	female	97	58	39	
	male	151	66	85	0.013
T stage	T1 + T2	146	88	58	
	T3 + T4	102	36	66	0.000
N stage	N0	233	121	112	
	N1	15	3	12	0.017
M stage	M0	207	113	94	
	M1	41	11	30	0.001
G stage	G1 + G2	111	72	39	
	G3 + G4	137	52	85	0.000
TNM stage	I + II	134	86	48	
	III + IV	114	38	76	0.000

### SAA1 Gene Promoter Region Is Hypomethylated in Patients With Advanced and Metastatic ccRCC

**Figure 5:**
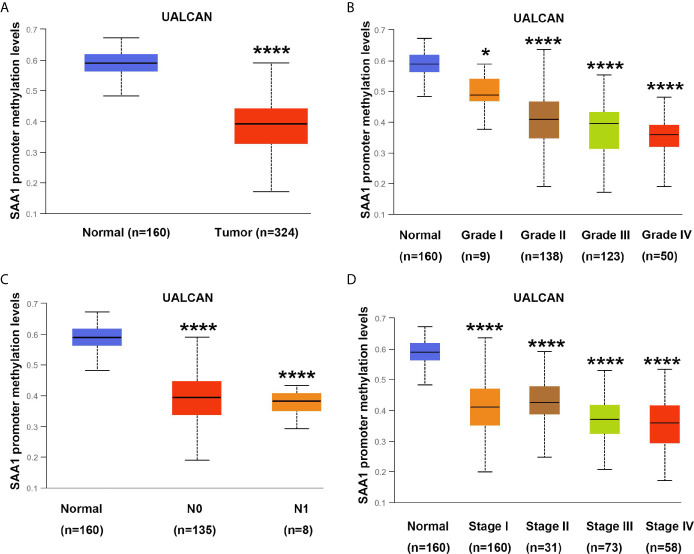
Analysis of methylation levels in SAA1 gene promoter region. **(A)** The methylation analysis of SAA1 gene promoter region in ccRCC samples from UALCAN database. **(B)** The methylation levels of SAA1 gene promoter region were compared in tumor grade from UALCAN database. **(C)** The methylation levels of SAA1 gene promoter region were compared in N stage from UALCAN database. **(D)** The methylation levels of SAA1 gene promoter region were compared in TNM stage from UALCAN database. (****P<0.0001, *P<0.05, compared with the respective control).

DNA methylation modification is an important component of epigenetics, which can silence the expression of methylated genes. To understand the cause of SAA1 overexpression in advanced and metastatic ccRCC, we analyzed the methylation status of SAA1 gene through the UALCAN online database. As shown in **Figure 5A**, the SAA1 gene was hypomethylated in ccRCC tissues, while it was hypermethylated in normal kidney tissues. Moreover, with the increase of tumor stage and grade, the degree of SAA1 gene methylation decreased accordingly, which means that the degree of SAA1 gene methylation was inversely related to the tumor’s stage (**Figures 5B–D**). These results indicate that the upregulation of SAA1 expression is due to the low methylation levels of the SAA1 promoter region in ccRCC and its methylation levels are inversely correlated with tumor stage and grade.

### SAA1 Possesses Diagnostic Value for Advanced and Metastatic ccRCC

**Figure 6:**
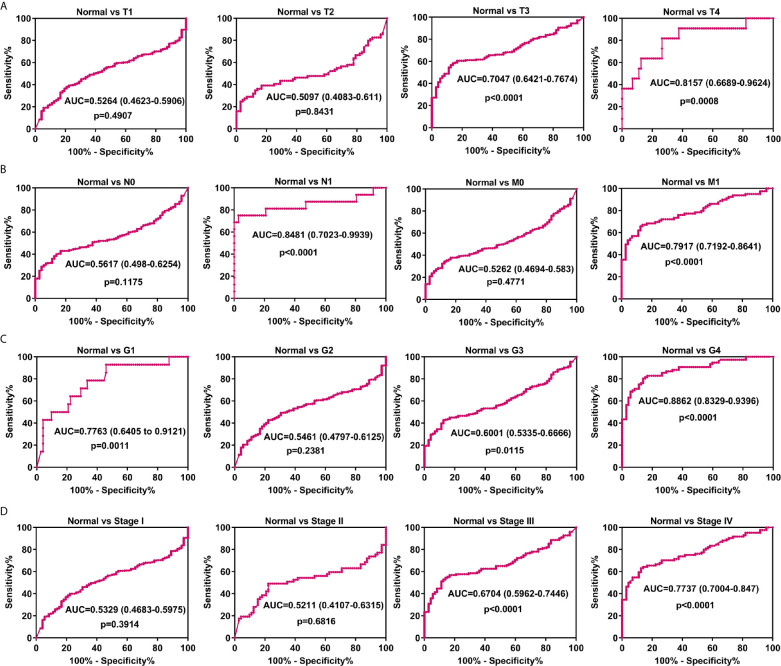
Analysis of the diagnostic value of SAA1 for advanced and metastatic ccRCC. **(A)** ROC curve analysis of the diagnostic value of SAA1 in patients with ccRCC at T stage. **(B)** ROC curve analysis of the diagnostic value of SAA1 in patients with ccRCC at N and M stages. **(C)** ROC curve analysis of the diagnostic value of SAA1 in patients with ccRCC at tumor grade. **(D)** ROC curve analysis of the diagnostic value of SAA1 in patients with ccRCC at TNM stage.

Biomarkers for tumor progression are still lacking in ccRCC patients. We found that SAA1 expression was significantly up-regulated at the mRNA and protein levels in patients with advanced and metastatic ccRCC. We wondered whether SAA1 could accurately diagnose patients with advanced and metastatic ccRCC. To determine the diagnostic value of SAA1, we performed ROC curve analysis between tumor tissues with different stage or grade and normal tissues. As shown in **Figures 6A–D**, high SAA1 levels could effectively distinguish advanced and metastatic ccRCC tissues from normal tissues (Normal/T4 (**Figure 6A**, AUC = 0.8157, p = 0.0008); Normal/N1 (**Figure 6B**, AUC = 0.8481, p < 0.0001); Normal/M1 (**Figure 6B**, AUC = 0.7917, p < 0.0001); Normal/G4 (**Figure 6C**, AUC = 0.8862, p < 0.0001); Normal/Stage IV (**Figure 6D**, AUC = 0.7737, p < 0.0001), but could not distinguish early ccRCC tissues from normal tissues. These results suggested that SAA1 could serve as a new diagnostic marker for patients with advanced and metastatic ccRCC. We wondered whether SAA1 predicts a similar prognosis in ccRCC patients with different stages and clinical parameters.

### SAA1 Possesses Prognostic Value for ccRCC Patients Regardless of Early and Advanced Tumors

**Figure 7:**
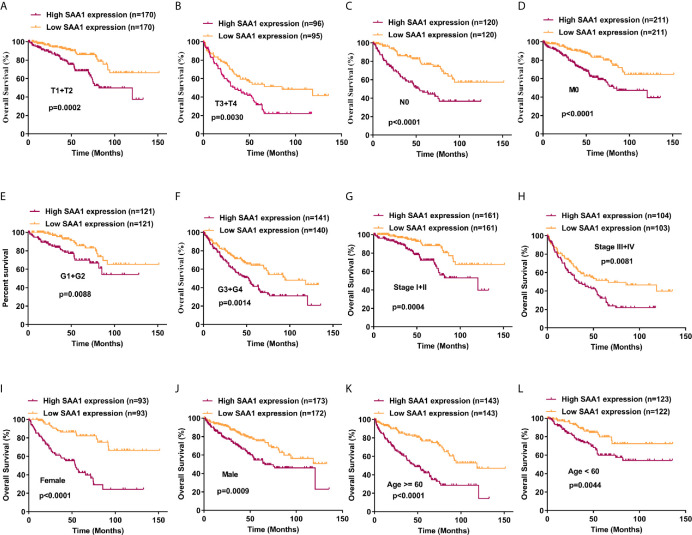
Analysis of the prognostic value of SAA1 for ccRCC. The ccRCC tissue samples from TCGA database were divided into low SAA1 expression group and high SAA1 expression group according to the median expression value of SAA1 mRNA level. The correlation between SAA1 expression and overall survival time of ccRCC patients was analyzed by Kaplan-Meier. (A-L) Overall survival analysis towards the expression of SAA1 mRNA was performed in subgroups of ccRCC patients: **(A)** T1+T2 stage, **(B)** T3+T4 stage, **(C)** N0 stage, **(D)** M0 stage, **(E)** G1+G2 stage, **(F)** G3+G4 stage, **(G)** Stage I+II, **(H)** Stage III+IV, **(I)** Female, **(J)** Male, **(K)** Age≥60 years, **(L)** Age<60 years.

Our previous results have confirmed that high expression of SAA1 predicts poor overall and disease-free survival in patients with ccRCC (**Figure 1B**). Moreover, our COX regression analysis found that SAA1 could be used as an independent prognostic factor for ccRCC (**Table 2**). We wondered whether SAA1 predicted a similar prognosis in ccRCC patients with different stages and clinical parameters. Therefore, we performed Kaplan Meier curve analysis towards the expression of SAA1 in ccRCC patients with different stages and clinical parameters (**Figures 7A–L**). Our results indicated that high SAA1 expression could serve as a potential prognostic factor for ccRCC patients with T1 + T2 stage (**Figure 7A**, p = 0.0002), T3 + T4 stage (**Figure 7B**, p = 0.0030), N0 stage (**Figure 7C**, p < 0.0001), M0 stage (**Figure 7D**, p < 0.0001), G1 + G2 stage (**Figure 7E**, p = 0.0088), G3 + G4 stage (**Figure 7F**, p = 0.0014), Stage I+II (**Figure 7G**, p = 0.0004), Stage III+IV (**Figure 7H**, p = 0.0081), Female (**Figure 7I**, p < 0.0001), Male (**Figure 7J**, p = 0.0009), Age ≥ 60 years (**Figure 7K**, p < 0.0001), Age < 60 years (**Figure 7L**, p = 0.0044).

**Table 2:** COX regression analysis between prognostic risk factors and overall survival of ccRCC patients.

Risk factors	Univariate analysis			Multivariate analysis		
	HR	95% CI	P value	HR	95% CI	P value
Age	1.803	1.318-2.468	0.000	1.579	1.147-2.175	0.005
Gender	0.948	0.697-1.290	0.736			
T stage	3.120	2.306-4.220	0.000	0.827	0.453-1.511	0.537
N stage	3.823	2.070-7.061	0.000	2.029	1.073-3.835	0.029
M stage	4.346	3.192-5.918	0.000	2.144	1.474-3.117	0.000
G stage	2.639	1.885-3.697	0.000	1.542	1.067-2.228	0.021
TNM stage	3.794	2.767-5.202	0.000	2.263	1.140-4.493	0.020
SAA1 expression	2.433	1.770-3.346	0.000	1.538	1.094-2.162	0.013

HR, Hazard ratio; CI, Confidence interval.

### The Protein Levels of SAA1 Were Examined in RCC Cell Lines and Tissues

**Figure 8:**
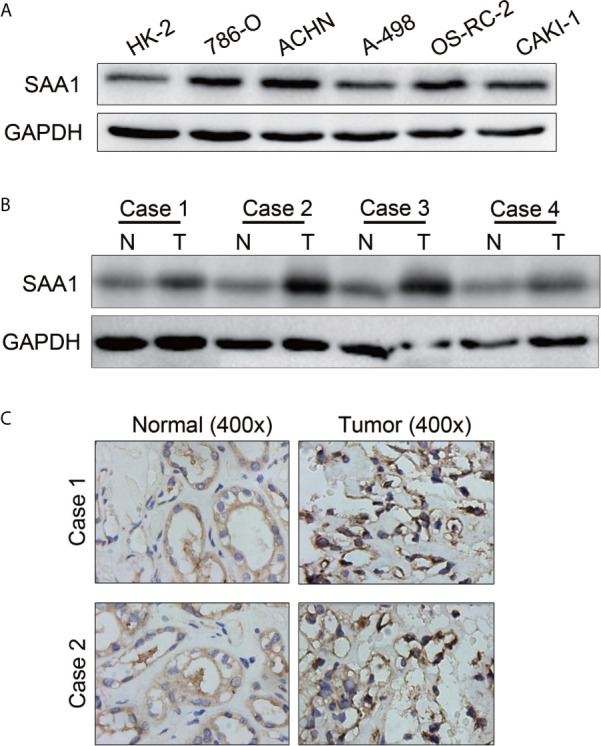
The protein levels of SAA1 in RCC cells and tissues. **(A)** Western blotting analysis of SAA1 expression levels in RCC cell lines (786-O, ACHN, A-498, OS-RC-2, Caki-1) and renal normal epithelial cells (HK-2). **(B)** Western blotting analysis of SAA1 protein levels in 4 pairs of ccRCC tissues (N, normal tissue; T, tumor tissue). **(C)** IHC analysis of SAA1 protein levels in normal renal tissues and ccRCC tissues.

To further confirm the results of the UALCAN, GEPIA and TCGA databases, SAA1 was subjected to western blotting in RCC cell lines and tissues. As shown in **Figures 8A, B**, the protein levels of SAA1 in RCC cell lines were significantly up-regulated compared with normal renal epithelial cell HK-2, and the protein levels of SAA1 in ccRCC tissues was also obviously overexpressed compared with adjacent normal tissues. The protein levels of SAA1 were also examined by immunohistochemistry (IHC) in paired ccRCC tissues, and the IHC results were consistent with the results of western blotting (**Figure 8C**). These results indicate that SAA1 is up-regulated in ccRCC cell lines and tissues, consistent with the results of UALCAN, GEPIA and TCGA database.

### SAA1 Knockdown Inhibits Migration and Invasion of RCC cells *In Vitro*

**Figure 9:**
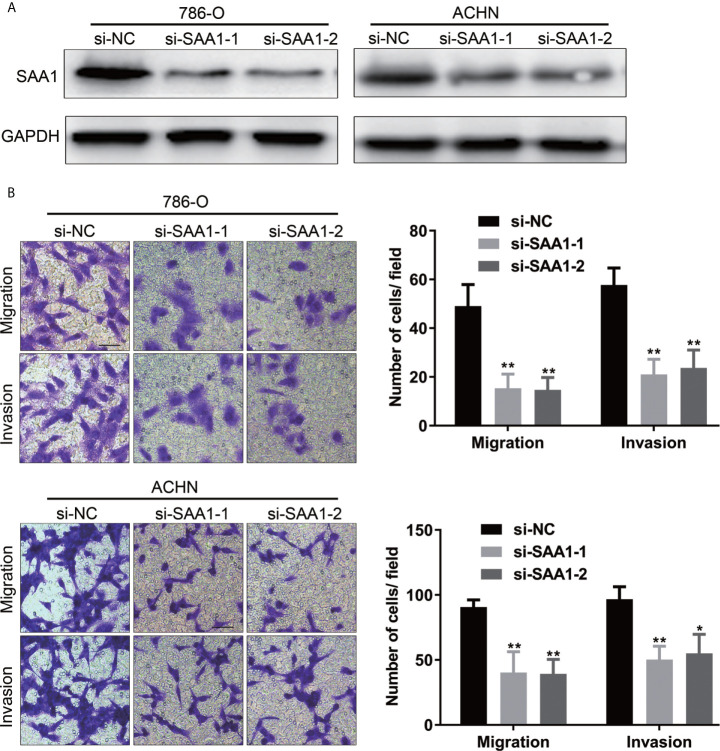
SAA1 knockdown significantly inhibits migration and invasion of RCC cells *in vitro*. **(A, B)** Transwell assays analysis of the effect of SAA1 knockdown on cell migration and invasion of 786-O and ACHN. (**P<0.01, *P<0.05, compared with si-NC group).

To investigate whether SAA1 affects the migration and invasion of RCC cells, we performed the transwell assays. As shown in **Figures 9A, B**, knockdown of SAA1 significantly impaired the migration and invasion capability of 786-O and ACHN cells. These results reversely suggest that SAA1 promotes migration and invasion of RCC cells.

## Discussion

The most common malignant tumor of the kidney is renal cell carcinoma (RCC), and ccRCC is the most common pathological subtype of RCC. The surrounding abundant vascularization causes ccRCC to grow easily and metastasize through the blood (28). It is reported that about 15% of RCC patients have distant metastases at the time of diagnosis (29). Moreover, treatment options for patients with advanced and metastatic ccRCC are limited and their survival prognosis is poor. Therefore, biomarkers screening for ccRCC progression is the key to improve the diagnosis and treatment of advanced and metastatic ccRCC. However, there is still a lack of clinical biomarkers for ccRCC progression and therapeutic targets for biomarkers.

SAA1 is currently recognized as an acute phase protein. When the body suffers from inflammation, trauma, surgery or advanced malignant tumors, the expression of SAA1 is significantly up-regulated (30). SAA1 has been reported to play a critical role in high-density lipoprotein metabolism and cholesterol homeostasis (11, 12). In addition, the role of SAA1 in tumor progression and its potential as a biomarker have also been extensively studied. For example, a large number of studies have reported that SAA1 promotes tumor progression and accelerates distant metastasis (31, 32). High SAA1 expression could be used as a potential biomarker for a variety of malignant tumors (18–20). Previous studies reported that SAA1 possessed the potential to become a prognostic marker of RCC (33, 34). Moreover, another study reported that serum SAA1 has been identified as a biomarker of distant metastases but not as an early tumor marker in RCC patients (21). These studies, unfortunately, did not detect the expression of SAA1 at the level of tumor cells and tissues. In addition, studies on SAA1 as a diagnostic and prognostic marker for advanced and metastatic ccRCC lack the support of extensive public research data.

In this study, we used three publicly published ccRCC GSE datasets to mine the top ten up-regulated genes and used the Wayne diagram to take the intersection in the three datasets. As a result, we screened out two candidate genes, SAA1 and CCL20. Next, we performed a prognostic analysis of these two genes using data from the TCGA database and found that only SAA1 possessed a predictive significance for ccRCC patients. So we used SAA1 as our target gene. To verify the accuracy of our screened genes, we analyzed SAA1 expression using publicly available TCGA, UALCAN and GEPIA databases. The results indicate that SAA1 is upregulated not only at the mRNA level but also at the protein level in ccRCC patients and exhibits higher expression levels in advanced and metastatic ccRCC. Next, we analyzed the reasons for the upregulation of SAA1 in ccRCC and found that the methylation levels of the promoter region of SAA1 gene were reduced, especially in advanced and metastatic ccRCC. ROC curve analysis found that SAA1 could only distinguish patients with advanced and metastatic ccRCC from the normal population, while Kaplan Meier curve analysis indicated that high SAA1 expression always predicted a worse prognosis regardless of tumor stage. Functionally, SAA1 knockdown significantly inhibits the migration and invasion of RCC cells *in vitro*.

Collectively, we found that SAA1 expression was up-regulated in kidney cancer tissues and its high expression was predictive of advanced tumor stage. In addition, SAA1 could serve as a biomarker for the diagnosis and prognosis of advanced and metastatic renal cell carcinoma at the tumor tissue level. Targeted SAA1 therapy might provide new treatment directions and good prognosis for patients with advanced and metastatic ccRCC.

Unsatisfactorily, there are some flaws in our research. Such as, the specific mechanism and molecular pathways of SAA1-mediated ccRCC metastasis remain unclear. The functions of SAA1 *in vivo* are still unclear. Moreover, our prediction data on tumor diagnosis and prognosis are mainly derived from cancer databases, and there is a lack of clinical and prognostic data for metastatic renal cell carcinoma patients, such as the International Metastatic Renal-Cell Carcinoma Database Consortium (IMDC) risk score. In addition, the SAA1 expression level in tissue specimens of metastatic renal cell carcinoma and its relationship with the prognosis of patients are still unclear. In subsequent experiments, we will continue to carry out relevant studies on these shortcomings. However, our study confirms that SAA1 expression is significantly up-regulated in advanced and metastatic ccRCC and can effectively distinguish patients with advanced and metastatic ccRCC from the normal population.

In summary, for the first time, we have demonstrated that SAA1 expression is significantly upregulated at the mRNA and protein levels in advanced and metastatic ccRCC. Moreover, SAA1 has great potential as a diagnostic and prognostic marker for advanced and metastatic ccRCC. In addition, targeted SAA1 therapy provides a new treatment and strategy for patients with advanced and metastatic ccRCC.

## Data Availability Statement

The datasets presented in the study are included in the article, further inquiries can be directed to the corresponding authors.

## Ethics Statement

The studies involving human participants were reviewed and approved by Huazhong University of Science and Technology. The patients/participants provided their written informed consent to participate in this study. Written informed consent was obtained from the individual(s) for the publication of any potentially identifiable images or data included in this article.

## Author Contributions

All authors contributed to the article and approved the submitted version.

## Funding

This study was supported by grant from National Natural Sciences Foundation of China (82002706, 81874090).

## Conflict of Interest

The authors declare that the research was conducted in the absence of any commercial or financial relationships that could be construed as a potential conflict of interest.
